# Liver Transplantation after Exertional Heatstroke-Induced Acute Liver Failure

**DOI:** 10.7759/cureus.768

**Published:** 2016-09-06

**Authors:** Faisal Inayat, Hafeez Ul Hasan Virk

**Affiliations:** 1 Department of Medicine, New York-Presbyterian Hospital, Weill Cornell Medical College, New York City, New York; 2 Department of Medicine, St. Luke’s-Roosevelt Hospital Center , Icahn School of Medicine at Mount Sinai, New York City, New York; 3 Memorial Sloan Kettering Cancer center, Memorial Sloan Kettering Cancer Center, New York City, New York

**Keywords:** heat stroke, acute liver failure, heat wave, liver transplantation

## Abstract

Exertional heatstroke (EHS) is a life-threatening disease characterized clinically by central nervous system dysfunction and severe hyperthermia. It frequently occurs among athletes, soldiers, and laborers. While cardiopulmonary symptoms are common in patients undergoing EHS, irreversible acute liver failure is a rarely described phenomenon. When managing cases of EHS complicated by acute liver failure, it is crucial to act promptly with aggressive total body cooling in order to prevent progression of the clinical syndrome. However, an urgent liver transplantation can be a therapeutic strategy when patients fail to improve with supportive measures.

## Introduction

Heatstroke (HS) is usually characterized by severe elevation of the core body temperature (above 40°C, 104°F) and central nervous system dysfunction [1-2]. The excessive heat production overwhelms the body's capacity to dissipate heat, usually in a hot and humid environment. Consequences can be far-reaching, potentially leading to multi-organ failure and possibly death [2]. 

Classical HS is generally caused by high environmental temperature, develops slowly over a few days, and can present with variable neuropsychiatric symptoms [1-2]. Exertional heatstroke (EHS) is usually observed in young individuals performing strenuous physical activity in hot environments. Certain prescribed or illicit drugs and genetic conditions are known risk factors [2]. However, EHS progressing to acute liver failure (ALF) is a rare clinicopathologic entity [3].

The present paper highlights that clinicians should maintain a high index of suspicion for prompt recognition of ALF in patients with EHS. Furthermore, the rationale for liver transplantation holds paramount importance in the appropriate clinical setting.

The patient agreed to participate and was explained the nature and objectives of this study, and informed consent was formally obtained. No reference to the patient's identity was made at any stage during data analysis or in the report.

## Case presentation

A 26-year-old Caucasian male was brought to the Mount Sinai St. Luke’s Hospital Emergency Department after he was found unresponsive in a car on a day with an outdoor temperature of 31°C (87.8°F). The patient had a Glasgow Coma Scale score of three upon arrival. He was intubated, ventilated, and resuscitated with intravenous fluid. The co-workers reported that the patient was doing manual labor when he complained of severe fatigue, and they put him in a car with the air conditioner turned on. However, after a couple of minutes, he was found unconscious. His past medical history was unremarkable.

Initial vital examination revealed a blood pressure of 128/63 mmHg, respiratory rate of 28, heart rate at 131 beats/min, core body temperature of 42.2°C (108°F), and oxygen saturation of 95% on room air. On examination, he was only responsive to noxious stimulus. No signs of head trauma or injury were found. In the resuscitation room, ice bags were placed all over his body, and an infusion of cold normal saline (0.9%) was initiated immediately. Cold water was poured on his body for active cooling. A temperature sensing Foley catheter was placed in the bladder. After two hours of continuous cooling techniques, his temperature dropped from 42.2°C to 38.2°C (108°F to 100.7°F). 

Biochemical investigations revealed levels of potassium (K) 5.4 mmol/L (normal: 3.5-5 mmol/L), bicarbonate 17 mmol/L (normal: 21-28 mmol/L), blood urea nitrogen (BUN) 23 mmol/L (2.5-7.1 mmol/L), serum creatinine 204 µmol/L (normal: <106 µmol/L), lactic acid 0.294 mmol/L (normal: 1.0-1.8 mmol/L), and creatine phosphokinase (CPK) 984 IU/L (normal: 60-174 IU/L). Liver function tests were within normal limits ruling out potential hepatic etiologies. Furthermore, the urine toxicology report came out negative for illicit substances. Computed tomography of the brain and abdomen was performed. It demonstrated diffuse brain injury, without focal intracranial pathology. Abdominal findings were significant for uniform low attenuation within the liver, suggestive of diffuse inflammation. There was no visible thrombus in the portal vein, inferior vena cava, or hepatic veins. Therein, after exclusion of other possible etiologies, the patient was diagnosed with EHS.

Subsequently, the patient was transferred to the intensive care unit (ICU). A significant improvement in his mental status was observed over a few hours. However, after 24 hours, blood started oozing from his intravenous access site, and the patient developed disseminated intravenous coagulation (DIC). His platelet level dropped to 26 x 10^9^/L(normal: 150-450 x 10^9^/L), international normalized ratio (INR) increased to 8.3 (normal: 0.8 to 1.2), and plasma fibrinogen decreased to 75 mg/dL (normal: 150-400 mg/dL). The patient was started on cryoprecipitate transfusion. After transfusion of ten units of cryoprecipitate, repeat laboratory studies revealed a fibrinogen of 82 mg/dL (normal: 150-400 mg/dL) and INR of 9.1 (normal: 0.8 to 1.2). Furthermore, aspartate aminotransferase (AST) and alanine aminotransferase (ALT) were unusually elevated to 9,129 IU/L (normal: 10 to 40 IU/L) and 6,679 IU/L (normal: 7 to 56 IU/L), respectively. The patient was administered 10 additional units of cryoprecipitate, with two units of fresh frozen plasma (FFP). Gastroenterology service recommended initiating intravenous N-acetyl cysteine (NAC).

Due to the continuously worsening condition of the patient, the medical board decided to transfer him to a nearby liver transplant center. The transplant team evaluated the status of the hepatic injury and deemed him a candidate for an urgent orthotopic liver transplantation (OLT). Thereby, the patient underwent an uneventful liver transplant with the donor being a close family member. Postoperatively, his liver function recovered over a 15-day period, and he was transferred from the ICU to the liver in-patient ward on post-transplant day 16. The patient had a relatively unremarkable postoperative recovery and was discharged from the hospital on postoperative day 21. He is now 12-months postoperative and continues to do well.

## Discussion

HS is a serious clinicopathologic entity that can lead to multiple organ failures. In the medical literature, there are numerous reports highlighting central nervous system dysfunction, rhabdomyolysis, renal failure, arrhythmias, cardiac failure, and DIC following HS [1-4]. However, ALF secondary to HS is relatively uncommon. The risk of EHS, a variant of HS, greatly increases in athletes, laborers, and soldiers with prolonged physical activity in high environmental temperatures and humidity [5]. 

The present case describes a healthy, physically fit young patient who developed EHS after strenuous activity performed under moderate heat conditions. The EHS in our patient subsequently complicated ALF. It prompts that the temperature measurement in a person who has been performing intense physical activity even in moderate weather conditions must be internally gauged, avoiding the interference of external factors [1].

Prompt cooling is the cornerstone of treatment, and the prognosis in EHS patients largely relies on this measure [1]. Numerous studies have demonstrated that if body temperature is reduced to less than 40.0°C (104°F) within 30 minutes after the loss of consciousness, the mortality rate markedly decreases [1]. There are authors who refer to this time frame as ''the golden half-hour'' in the realm of EHS [1]. Antipyretics counteract the change in the hypothalamic set-point caused by pyrogens [1-2]. Therefore, the efficacy of antipyretics has been controversial in the management of EHS.

The exact pathogenesis of underlying liver failure in EHS is yet to be determined, but it is hypothesized that a state of hypoxic hepatitis ensues secondary to ischemic changes. Ischemia may develop due to systemic hypoperfusion, thrombosis of related blood vessels, or cardiac failure due to increased overload, which may lead to intrahepatic circulatory failure [1]. It is believed that massive liver cell necrosis results from thermal shock, circulatory disruption, endotoxemia (heat sepsis), high blood concentration of cytokines, and acute-phase proteins [1-3].

Correct identification of ALF may get delayed owing to the neurological impairment in patients with EHS. Major causes of ALF must be ruled out first, including hepatitis infection, drug-induced liver injury, severe sepsis, primary or metastatic malignancy, mushroom ingestion, and various metabolic diseases [6]. If the etiology remains unknown after extensive investigations, histopathologic analysis after a careful liver biopsy should be considered. It has been observed that EHS patients with encephalopathy deteriorate rapidly; therefore, such patients should be transferred to the ICU immediately, and contact with a transplant center should be made in good time [7].

On admission, sufficient intravascular volume maintenance is recommended in the management of ALF. If hypotension is present, the initial treatment should be intravenous normal saline. ALF patients are on the verge of developing hypoglycemia so continuous glucose monitoring is of immense importance. Nutritional support, including 1.0–1.5 g of enteral protein per kilogram per day, should be carried out due to high-energy expenditure in these patients [6]. When renal dysfunction develops in ALF, it usually gets resolved on its own without specific management. Therefore, in our opinion, the treatment focus should be ALF instead of renal dysfunction in such cases. 

Although most cases do not bleed because of an adequate balance between anticoagulant loss and procoagulant factor synthesis, coagulation variables should be monitored at regular intervals [6]. Prophylactic transfusion of plasma is not recommended. In the absence of hemorrhage, transfusion would conceal trends in the INR, which is an important marker of prognosis in cases with EHS presenting with ALF [6-7]. Volume overload or transfusion-related acute lung injury can also develop in such cases.

Currently, there are no well-validated prognostic tools that predict the need for or survival with OLT for HS. Several cases of EHS-induced liver failure have been successfully managed with non-operative treatments [4]. However, the prognosis of patients presenting with acute liver failure from EHS is largely unpredictable; therefore, indications for liver transplantation in this specific setting are unclear. Hence, patients with non-paracetamol ALF with progressive coagulopathy, as in this presented case, should be discussed with a transplant center.

There are five reported cases of HS-induced fulminant hepatic failure requiring super-urgent OLT, but the results were not particularly impressive [8]. However, in a recent study conducted in Ireland, total hepatectomy and liver transplantation as a two-stage procedure for fulminant hepatic failure is deemed as a safe procedure in exceptional circumstances, including HS [9]. Therefore, transplantation medicine requires further research in this area for evidence-based management of patients with HS-induced ALF.

In our case, the decision to proceed with an urgent OLT was based on the patient’s rapid clinical deterioration, which did not respond to medical treatment and was associated with gradually worsening hepatic failure. Therefore, we took a timely decision for OLT, and the life of the patient was saved. Ultimately, he made a complete recovery soon after the OLT, with resolution of the liver and central nervous system dysfunction.

Heatwaves are associated with marked, short-term increases in mortality owing to HS. From this perspective, the consequences have been far more detrimental in Southern Europe and Southeast Asia. In a heatwave, temperatures stay considerably more elevated than usual. Increased humidity often exacerbates the weather conditions, which further contributes to HS and may result in an exponentially increased heat-illness-related mortality. Therefore, climate change, an aging population cohort, and certain medications may culminate in an increased number of heat-related disorders, including ALF.

## Conclusions

EHS with liver involvement to the extent of ALF is a rare clinical event, which may represent a major therapeutic dilemma. The option of OLT may be considered in patients with biochemical and clinical evidence of massive hepatocellular damage. However, further clinical studies are required to broaden the scope of our knowledge on this issue and to frame guidelines to standardize the care of patients with EHS-associated ALF. This may help to select patients early in their presentation who would benefit from OLT, leading to better clinical outcomes.
